# Comparison of Retinal Imaging Techniques in Individuals with Pulmonary Artery Hypertension Using Vessel Generation Analysis

**DOI:** 10.3390/life12121985

**Published:** 2022-11-28

**Authors:** Mariana DuPont, John Hunsicker, Simona Shirley, William Warriner, Annabelle Rowland, Reddhyia Taylor, Michael DuPont, Mark Lagatuz, Taygan Yilmaz, Andrew Foderaro, Tim Lahm, Corey E. Ventetuolo, Maria B. Grant

**Affiliations:** 1 Department of Optometry and Vision Science, School of Optometry, University of Alabama at Birmingham, Birmingham, AL 35294, USA; 2 Heersink School of Medicine, University of Alabama at Birmingham, Birmingham, AL 35294, USA; 3 Department of Political Science and Public Administration, University of Alabama at Birmingham, Birmingham, AL 35294, USA; 4 Research Computing, University of Alabama at Birmingham, Birmingham, AL 35294, USA; 5 Department of Osteopathic Medicine, The Philadelphia College of Osteopathic Medicine, Philadelphia, PA 19131, USA; 6 Redline Performance Solutions, Ames Research Center, National Aeronautics and Space Administration, Moffett Field, Mountain View, CA 94043, USA; 7 Division of Ophthalmology, Department of Surgery, Alpert Medical School of Brown University, Providence, RI 02903, USA; 8 Division of Pulmonary, Critical Care and Sleep Medicine, Department of Medicine, Alpert Medical School of Brown University, Providence, RI 02903, USA; 9 Department of Medicine, Division of Pulmonary, Critical Care and Sleep Medicine, National Jewish Health, Denver, CO 80206, USA; 10 Department of Medicine, Division of Pulmonary Sciences and Critical Care Medicine, University of Colorado, Aurora, CO 80045, USA; 11 Rocky Mountain Regional VA Medical Center, Aurora, CO 80045, USA; 12 Department of Health Services, Policy and Practice, Brown University School of Public Health, Providence, RI 02903, USA

**Keywords:** retinal imaging, fluorescein angiography, VESGEN, image processing, vascular segmentation

## Abstract

(1) Background: Retinal vascular imaging plays an essential role in diagnosing and managing chronic diseases such as diabetic retinopathy, sickle cell retinopathy, and systemic hypertension. Previously, we have shown that individuals with pulmonary arterial hypertension (PAH), a rare disorder, exhibit unique retinal vascular changes as seen using fluorescein angiography (FA) and that these changes correlate with PAH severity. This study aimed to determine if color fundus (CF) imaging could garner identical retinal information as previously seen using FA images in individuals with PAH. (2) Methods: VESGEN, computer software which provides detailed vascular patterns, was used to compare manual segmentations of FA to CF imaging in PAH subjects (n = 9) followed by deep learning (DL) processing of CF imaging to increase the speed of analysis and facilitate a noninvasive clinical translation. (3) Results: When manual segmentation of FA and CF images were compared using VESGEN analysis, both showed identical tortuosity and vessel area density measures. This remained true even when separating images based on arterial trees only. However, this was not observed with microvessels. DL segmentation when compared to manual segmentation of CF images showed similarities in vascular structure as defined by fractal dimension. Similarities were lost for tortuosity and vessel area density when comparing manual CF imaging to DL imaging. (4) Conclusions: Noninvasive imaging such as CF can be used with VESGEN to provide an accurate and safe assessment of retinal vascular changes in individuals with PAH. In addition to providing insight into possible future clinical translational use.

## 1. Introduction

The human eye offers a window into systemic diseases and disease progression [1,2,3]. There is, thus, clinical demand for imaging modalities of the retina with the ability to detect changes in disease progression safely and effectively [1,2,4]. Early detection of alterations in retinal vessels allows for interpretation of the overall health of systemic blood vessels and potentially allows for early treatment of retinal diseases to prevent long-term damage, including blindness. Diabetes and hypertension, for example, can cause alterations in the retinal arteries, such as vascular narrowing and arteriovenous occlusion, leading to retinal pathology [5,6,7].

Fluorescein angiography (FA) is a widely used invasive procedure involving the injection of sodium fluorescein to visualize retinal vasculature. It has been associated with an adverse event rate ranging from 1–22% [8]. The most commonly reported are nausea, vomiting, gastrointestinal upset, and urticaria, with reports of more severe complications including anaphylaxis, cardiac events, tonic-clonic seizures, and death [8,9]. According to a recent comprehensive literature review, 11 reported deaths have been associated with FA, amounting to an estimated 1:220,000 to 1:100,000 death rate [10,11,12]. Moreover, individuals with underlying systemic arterial hypertension, pulmonary artery hypertension (PAH), diabetes, sickle cell disease, or allergy history may be at increased risk of adverse events due to compromised kidney function. Importantly, retinal pathology has been associated with these systemic conditions [13].

Given these concerns, there is a need for noninvasive methods to detect retinal vascular changes without sacrificing image quality. Color fundus (CF) imaging is typically performed before FA, and CF produces a colorized image using a fundus camera under the illumination of white light. Unlike the adverse events associated with FA imaging, there have been no known reports of photic injuries from standard fundus photography [14]. CF is time-efficient and user-friendly, typically completed within minutes during routine clinic visits. In contrast, FA takes up to an hour to perform at a greater cost.

Previous studies have reported comparisons between the efficacy of FA and CF. Studies have revealed almost equivalent results between FA and CF with regard to the detection of a critical retinal zone in infants with retinopathy of prematurity along with the detection of peripheral diabetic retinal changes, supporting CF as a vital retinal imaging modality. However, previous reports lack information regarding changes in vessel generation which can be assessed using a novel program called vessel generation (VESGEN) analysis [15,16].

VESGEN 2D analysis is a NASA-based application with a user-interactive image J plugin. Previously VESGEN has been used in other studies [5], including studies using VESGEN 2D to investigate the progression of diabetic retinopathy.

Color fundus vessel segmentation is a notoriously difficult image-processing task. Numerous studies have been published with varying degrees of success in attempting to accomplish this [17,18]. The second major purpose of this work was to develop an appropriate method for reducing VESGEN image preparation time by comparing deep learning to standard image processing. Image segmentation is the combination of algorithms that detect, refine, and extract aspects of interest from CF pictures. Such techniques must be carefully designed, although only a few CF pictures are required for optimization. This study intends to demonstrate that in individuals with PAH, CF may identify vascular abnormalities in a manner equivalent to FA.

## 2. Materials and Methods

### 2.1. Clinical Study

The cohort of subjects for this study were individuals who have been confirmed to carry the diagnosis of the rare World Health Organization (WHO) Group 1 PAH. Informed consent was obtained from all subjects involved in the study. In a previously published report, we compared these PAH subjects to controls and the relationship between retinal parameters obtained by FA and clinical measures of PAH severity [19]. PAH subjects were recruited from the Rhode Island Hospital Pulmonary Hypertension Center. Each subject underwent both FA and CF on the same day. The Institutional Review Boards approved this study at Rhode Island Hospital (Study #411516).

### 2.2. Image Acquisition and Processing

CF images of the subjects’ retinas were performed by retinal photographers at Rhode Island Hospital. Images were acquired with a 55-degree field of view and resolution of 2392 by 2048 pixels, and processed, traced, and analyzed using 2019 and 2020 Photoshop Adobe Creative Cloud. To ensure uniformity among images, the final analysis of images was processed by a single individual, masked to the identity of the subjects and disease severity. Using the physiological vascular branching rules previously described, the CF image was used to define the difference between arteries and veins [5]. FA images of the same eye were acquired similarly. All images were reviewed and signed off by our NASA VESGEN expert before any binarization of the images and input in VESGEN. Three FA images were excluded from the analysis due to poor image quality.

### 2.3. Vascular Quantification

The VESGEN software is a JAVA-based interactive vascular analysis platform that is globally available from NASA (https://software.nasa.gov/search/software/vesgen, accessed on 15 May 2019) and operates as a complex plug-in to ImageJ software (National Institutes of Health, Bethesda, MD, USA) [20,21]. VESGEN 2D examines a binary image of a 2D vascular structure using the User-selected Vascular Morphology.

Some output provided by VESGEN that was also used for this study include fractal dimension defined as the ratio of statistical index of complexity or the structural changes occurring as the vasculature changes from large vessels to small vessels, vessel area density defined as vascular area divided by the region of interest, tortuosity define as the length of vessel divided by the distance between the endpoints of the vessels, and number of vessels defined as the number of vessels identified based on each generation.

The quantification of the retinal findings are given in pixel units. Highly sophisticated algorithms define branch boundaries and assign proper branching generations in order to study vascular trees. Output measurements are delivered in the form of a one-page summary document as well as an extensive multi-page measurement confirmation document.

Manuel segmentation was performed for both FA and CF images prior to binarization. Binary (black/white) images of the blood vessels were obtained in parallel from manual preparation and deep learning processing. The region of interest (ROI) was obtained by image processing. These images were imported into VESGEN, automatically mapping and quantifying various vascular parameters. Parameters of interest included the following: (1) Tortuosity (Tv; length of vessel divided by the distance between the two endpoints of the vessel), (2) fractal dimension (Df; a ratio for determining the complexity of the given measurement), and (3) vessel area density (Av; density of total vascular area, Av = vascular area/ROI). VESGEN assigned vessels generations. Macrovessels were defined as generations 1 to 5 and microvessels were defined as generations 6 and greater.

### 2.4. Deep Learning

The deep learning approach used was RVGAN, short for retinal vessel generative adversarial network (GAN), programmed in Python using TensorFlow. More details on model training and operation are available at https://github.com/SharifAmit/RVGAN, accessed on 6 January 2020 [22,23,24]. We used an instance of the RVGAN model pretrained on the STARE dataset, provided by its creator. This pretrained model was selected over the DRIVE and CHASE alternatives due to our dataset’s better “to-the-eye” qualitative performance. For our dataset, RVGAN models pretrained with CHASE and DRIVE produced unusable images. Unusable here means most of the vessels were not segmented. We used the model pretrained with STARE because it did not exhibit this issue.

Images in the STARE dataset had 18.5 pixels per degree of field of view (FOV), while the images used in this study had 40.9 pixels per FOV degree. Fundus images were scaled down using linear interpolation to match the STARE dataset resolution for correct segmentation. The output binary images were scaled up linearly and thresholder at 50% to match the original input image sizes, and for comparison with the traditional segmentation. Arteries were manually separated from the output binary images for analysis.

Custom model evaluation code was written in Python to handle scaling and application of the pretrained model using libraries from the SciPy stack. The resulting binary segmentation images were cleaned in MATLAB R2021a by removing small, connected components. The fundus photograph region of interest (ROI) masks was eroded by 3 pixels and applied to the binary images to trim artifacts caused by model edge effects. A border of 3 pixels around the edge of the binary images was removed to trim the remaining edge artifacts. Arteries were manually separated from binary images.

### 2.5. Statistical Analysis

The Bland–Altman plot was used as it compares two measurements of the same variable. The *X*-axis represents the mean of two measurements, and the *Y*-axis represents the difference between the two measurements [25]. Plotting difference versus mean aids in discovery of possible relationships between measurement inaccuracy and real value. Left and right eyes were grouped separately. Agreement between biometrics was examined by eye (left vs. right) using Bland–Altman plots with 2 (red) and 3 (green) standard deviation reference bounds using SAS Software 9.4 (Cary, NC, USA). If points are scattered above and below zero (mean black line) then there is no persistent bias toward one imaging modality over the other. In contrast, if values generally increase or decrease then there is evidence that the measures are not concordant. Bland–Altman analyses provide evidence for agreement and thus cannot be used to determine if one method is better than another (i.e., because there is no gold standard).

## 3. Results

### 3.1. Subject Characteristics

FA and CF images of 15 eyes from 9 individuals were compared. Detailed clinical characteristics have been previously described [19]. Mean age of subjects was 50, with the youngest 26 and the oldest 72 years old. Eight of the nine subjects (89%) were females, and seven were Caucasian (77%).

### 3.2. Color Fundus Imaging of Retinal Vascular Phenotype in PAH

**Figure 1:**
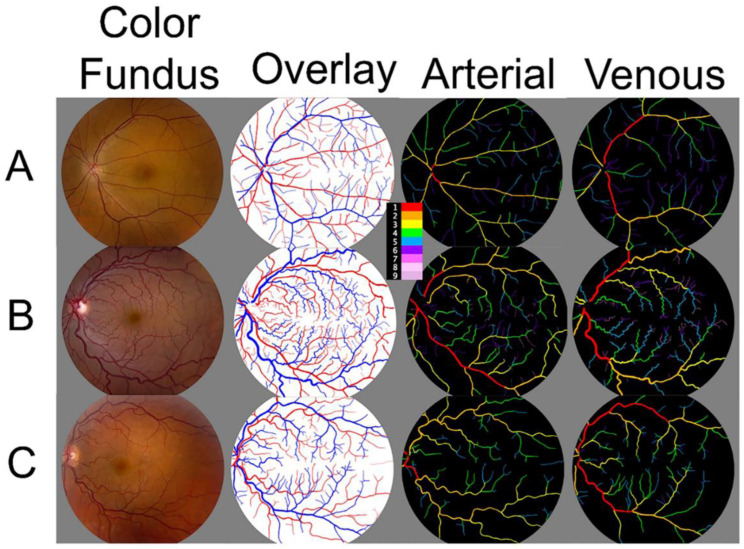
Representative output image of VESGEN analysis of the retinal vessels from PAH subjects. VESGEN imaging maps with a 55-degree imaging resolution of the right retina from a (**A**): 51-year-old female PAH subject; (**B**) 36-year-old female PAH subject; (**C**) 72-year-old female PAH subject. First Column: Color fundus (CF) output image. Second column: Overlap of arteries (red) and veins (blue) generated manually. Third and fourth columns: Branching generation of arteries and veins, respectively, generated by VESGEN output. Legend (center) identifies branching generations 1–9.

CF images (Figure 1; first column) with 55-degree field of view were processed, and binary images for VESGEN input were created to identify regions of interest. CF images were also used to identify arteries and veins, resulting in an overlapping image where arteries are red, and veins are blue (Figure 1; second column). The generational summary was analyzed separately for arteries (Figure 1; third column) and veins (Figure 1; 4th column). Increasing disease severity is shown from row A to row C.

### 3.3. Manual Segmentation: CF vs. FA

To determine whether CF images could be used as a viable alternative to FA imaging in individuals with PAH, we compared manual segmentation of total (artery and veins) vessels, and artery only, in both Tv and Av, using FA and CF images along with the microvessels of total (arteries and veins) vessels. Tv and Av have previously been shown to indicate the progression of retinal pathology in diabetic retinopathy [5] and spaceflight associated neuro-ocular syndrome (SANS) [26,27]. The left eye and right eye were separated for VESGEN analysis. For manually segmented images, total vessels (both arteries and veins) and arteries only were each compared.

**Figure 2:**
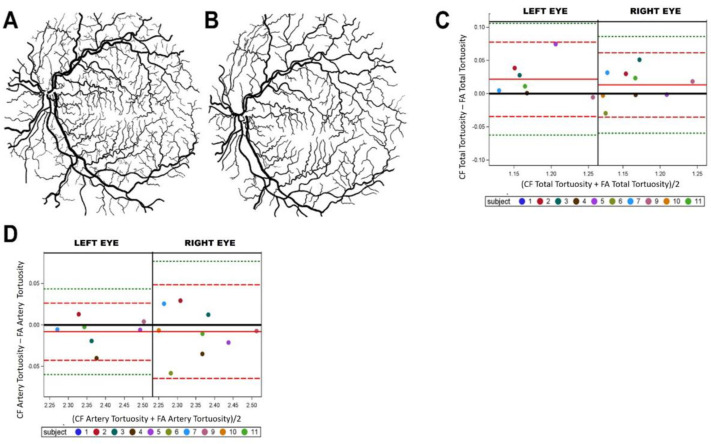
Fluorescein angiography and color fundus comparisons of vascular tortuosity. Manual segmentations of fluorescein angiography (FA) binary images (**A**,**B**) color fundus (CF) prepared manually from the retina of a 45-year-old female with PAH. Right and left eyes were split on Bland–Altman plot where FA VESGEN output was compared to CF VESGEN output for total tortuosity, showing a scatter of points suggesting similar results between imaging methods (**C**), and total artery tortuosity showing scatter of points suggesting similar results between image methods (**D**). The mean of the measures (*x* axis) and the difference of the measures (*y* axis). The reference line of no difference at 0 (solid black), a reference line at the mean of the difference in solid red (observed actual mean), a reference line at +/−2SD of the mean of the difference in dashed red, and a reference line at +/−3SD of the mean of the differences in dashed green (**C**,**D**).

A prepared manually segmentation from the retina of a 45-year-old female with PAH in shown in Figure 2A,B. The manual processing of FA images (Figure 2A) and manual processing of CF images (Figure 2B) were compared in all individuals. To better understand if Tv, a known hypertension characteristic, can be identified using CF similarly to using FA. Bland–Altman plots were used for comparison. Tv total vessels (arteries and veins) and arteries only, the key component of the vasculature affected by PAH were examined. Tv output between CF and FA showed an equal scattering of points both above and below the red solid line (mean), suggesting both imaging techniques (CF and FA) can produce similar levels of Tv analysis in both higher and lower ranges (Figure 2C, 0.021 ± 0.28 vs. 0.013 ± 0.024). When comparing FA and CF with respect to arteries only, some points are scattered below and above the mean line (red solid line), also suggesting that both imaging methods result in similar Tv (Figure 2D, −0.008 ± 0.016 vs. −0.007 ± 0.028). These findings showed that manually segmented CF and FA fundus images contain similar levels of vessel tortuosity.

**Figure 3:**
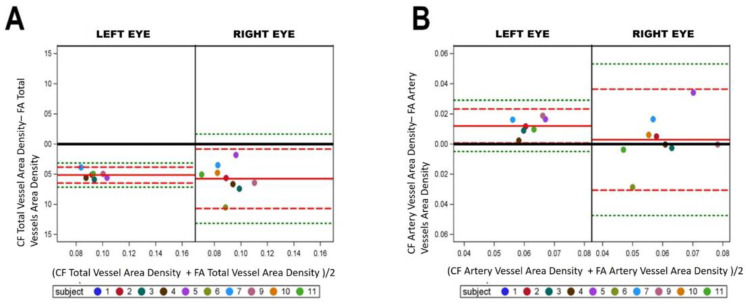
Fluorescein angiography and color fundus comparisons in vessel area density. (**A**) Left and right eyes were split on Bland–Altman plot where fluorescein angiography (FA) VESGEN output was compared to color fundus (CF) VESGEN output for total vessel area density. Scattered points for both left and right eye data suggests no difference between imaging methods. (**B**) Total artery vessel area density with scattered points suggests no difference between imaging methods with points gather near mean. Plots show similarities between FA and CF imaging among total Av and total artery Av suggesting that CF can be used as a safer method for vascular change monitoring. The mean of the measures (*x* axis) by the difference of the measures (*y* axis). The reference line of no difference at 0 is the solid black, a reference line at the mean of the difference in solid red (observed actual mean), a reference line at +/−2SD of the mean of the difference in dashed red, and a reference line at +/−3SD of the mean of the differences in dashed green.

To identify angiogenesis and proliferation by vascular density via the ROI Vessel area density (Av) was examined. As with above, the Bland–Altman plots suggest comparable Av results for total vessels across both imaging modalities (Figure 3A, −0.051 ± 0.006 vs. −0.057 ± 0.024). The same comparable Av results held for artery-only (Figure 3B, 0.012 ± 0.005 vs. 0.002 ± 0.0016). These findings support the idea that manually segmented CF and FA fundus images contain similar vascular densities.

**Figure 4:**
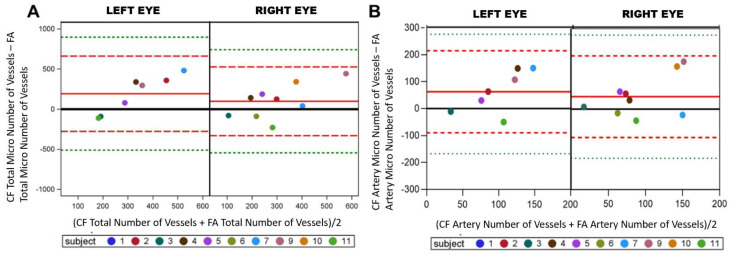
Fluorescein angiography and color fundus comparisons in microvessel number. Left and right eyes were split on Bland–Altman plot where fluorescein angiography (FA) VESGEN output was compared color fundus (CF) VESGEN output for total number of microvessels (**A**) and total number of microvessel of the arteries (**B**). Plots show differences between FA and CF imaging in microvessels of total vessels due to the linear scattering of the points. The mean of the measures (*x* axis) and the difference of the measures (*y* axis). The reference line of no difference at 0 (solid black), a reference line at the mean of the difference in solid red (observed actual mean), a reference line at +/−2SD of the mean of the difference in dashed red, and a reference line at +/−3SD of the mean of the differences in dashed green.

Microvessels are commonly affected in many systemic vascular diseases, so we examined and compared the number of microvessels (G ≥ 6). For both total microvessels and arteries only, a trend is apparent in the Bland–Altman plots for left eyes, suggesting FA and CF imaging are not comparable. With right eyes, there is a more scattered distribution of points about the mean indicating better possible concordance across imaging modalities. Further, the right eye means (red lines) are closer to zero (black lines) in total microvessels (Figure 4A, −193.42 ± 234.85 vs. 98.22 ± 214.29), and in artery microvessels (Figure 4B, −62.57 ± 77.71 vs. 46.56 ± 77.69).

### 3.4. CF Manual Segmentation vs. CF Deep Learning Segmentation

**Figure 5:**
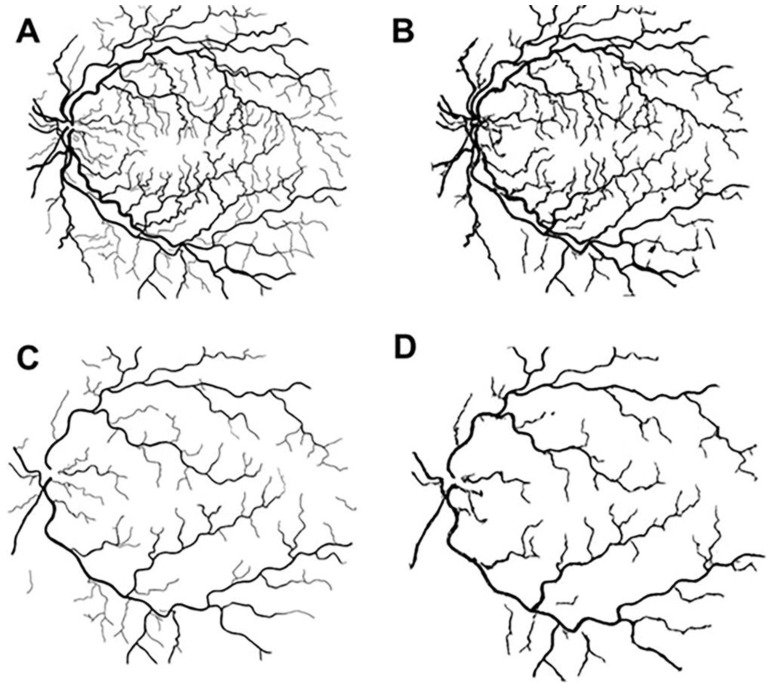
Binary images of manual and deep learning. CF binary images prepared (**A**) manually and by (**B**) deep processing using the retina of a 45-year-old female with PAH. CF binary images of the artery prepared (**C**) manually and by (**D**) deep processing using the retina of a 45-year-old female with PAH.

Manual (Figure 5A) and deep learning segmented (Figure 5B) images were compared. The model is only trained for use with CF images, so FA images were not compared. Due to the inability of our deep learning’s approach to separate arterial and venous structures, total vasculature was first segmented using deep learning (Figure 5C). Following total vessel segmentation our NASA trained VESGEN expert manually separated arterials from total vessels for comparison of arterials only (Figure 5D).

**Figure 6:**
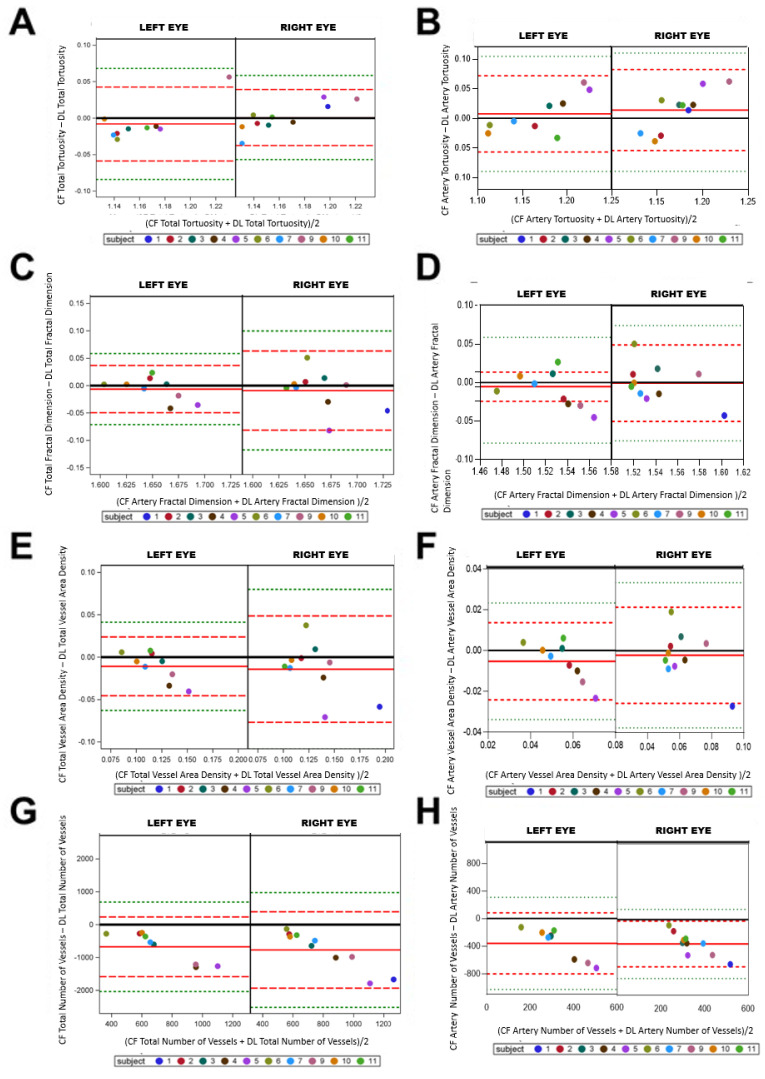
Manuel and deep learning comparisons. Right and left eyes were split on Bland–Altman plot where color fundus (CF) VESGEN output was compared deep learning VESGEN output for total tortuosity (**A**), arterial tortuosity (**B**), total fractal dimension (**C**), arterial fractal dimension (**D**), total vessel area density (**E**), arterial vessel area density (**F**), total number of vessels (**G**) and arterial number of vessels (**H**). Plots show difference between FA and CF imaging among total vessels (Tv) (**A**), total tortuosity (**C**), and total number of vessels due to the liner scattering of the points. Plots show similarities between FA and CF imaging among total Df. This suggesting CF can be used as a safer method for vascular change monitoring. The mean of the measures (*x* axis) by the difference of the measures (*y* axis). The reference line of no difference at 0 is the solid black, a reference line at the mean of the difference in solid red (observed actual mean), a reference line at +/−2SD of the mean of the difference in dashed red, and a reference line at +/−3SD of the mean of the differences in dashed green.

Tv of manual and deep learning segmented images was compared. Both eyes showed more points below the mean in the Bland–Altman plot suggesting one had lower Tv (Figure 6A, −0.008 ± 0.025 vs. 0.0008 ± 0.019). The artery only Bland–Altman plots showed a greater number of points above the mean, suggesting one image method had greater Tv (Figure 6B, 0.007 ± 0.032 vs. 0.013 ± 0.034). Comparison of Tv between manual CF and CF deep learning segmentation using Bland–Altman plots showed a downward trend suggesting manual and deep learning segmentation are not comparable for Tv.

Fractal dimension (Df) proved more comparable between manual and deep learning images. The Bland–Altman plots for total vessels showed a scattered distribution of points, with most points near the mean (solid red line) in the left and right eye. This suggests both imaging methods can detect Df changes among the entire image (Figure 6C, −0.0063 ± 0.021 vs. −0.0089 ± 0.036). Similarly for arteries, points were scattered about the mean, suggesting similarity between manually CF and deep learning. However, the left eye has an apparent linear relationship, suggesting poor concordance of the left eye manual and deep learning segmentation in the right eye for arteries (Figure 6D, −0.0098 ± 0.023 vs. −0.00084 ± 0.025).

Manual and deep learning segmentation were compared for vessel area (Av). Av showed an unfavorable comparison, with most points falling above the mean, suggesting poor concordance, with one method resulting in higher Av (Figure 6E, −0.0108 ± 0.017 vs. −0.014 ± 0.031). The same results were observed for Av of artery only images (Figure 6F, −0.0052 ± 0.0096 vs. −0.0021 ± 0.011).

Last we compared deep learning’s ability to identify the same number of vessels as manual preparation. Most points are gathered above the mean (red line), suggesting that one image method results in a higher number of vessels than the other image method. Thus, the imaging methods did not result in similar output data (Figure 6G, −769.4 ± 581.91 vs. −674.66 ± 453.41). Arterials showed a poor similarity between manual and deep learning process using CF (Figure 6H, −359.7 ± 225.6 vs. −352.9 ± 169.5). In the comparison of Av and number between manual CF and CF deep learning segmentation using Bland–Altman plots showed a trend suggesting the failure of the two image methods to provide similar density and number of vessels.

## 4. Discussion

In this study, we provide further validation that a non-invasive technique may be useful for early detection or serial monitoring in PAH individuals. This study aimed to compare two imaging techniques, FA and CF imaging, and determine whether CF provided comparable clinically relevant results. VESGEN, compared to other vasculature tracing program, has increased sensitivity to detect smaller changes and allows examination of arteries and veins separately. Unlike other vasculature tracing programs, VESGEN can examine both large and small vessels independently. To our knowledge our study is the first to use VESGEN to compare FA and CF images as until now VESGEN has only been used with FA images.

Using VESGEN to analyze the images, CF images detect retinal vascular changes similar to FA in PAH subjects. We show that CF is able to detect retinal Tv and changes in total vascular area (Av = vascular area/ROI), two parameters that are associated with disease severity in PAH subjects [19].

Our results suggest that similar clinical information can be obtained from CF and FA. However, CF has the added advantage of being safer for patients. While FA and CF were not concordant in identifying microvessels numbers, FA and CF resulted in similar T_v_ and A_v_. Due to the inability in CF to substitute for FA in monitoring microvascular diseases, using CF for diseases such as diabetic retinopathy may not be feasible. The second main finding of this study is that we identify deep learning as an approach to reduce image preparation time. Image processing segmentation combines algorithms that identify, refine, and extract features of interest from CF images. These findings may provide insight into using retinal vascular changes as evaluated by VESGEN to diagnosis retinal diseases earlier and may be adapted for use in telemedicine [28].

The difference between left and right eyes was an unexpected discovery. The left eye often resulted in poor concordance in various vascular patterns between image methods, whereas the right eye had stronger concordance. Notably, a 2018 [29] investigation in early-stage systemic hypertension alterations in the retina intended to investigate choriocapillaris vasculature ocular/systemic variables. The eyes of 361 healthy people and 206 people with systemic hypertension were investigated in this study. They discovered that the right eye’s choriocapillaris vascular density was substantially higher than the left eye’s. One theory proposed to explain the difference is the difference in origin of the left and right ophthalmic arteries. While both ophthalmic arteries originate from their respective internal carotids, the left common carotid originates from the aorta while the right common carotid artery comes from the brachiocephalic trunk. An alternative explanation for these changes between the left and right eye may simply be due to the imaging protocol. While both eyes were dilated, our protocol did not specify which eye should be examined first. Thus, there may be a bias towards better images in the eye that is first examined as the experimental subject may be less tired and more cooperative with the procedure allowing for better visualization.

In addition, the Bland-Alman analysis could only tell if the two methods (CF and FA) were similar but not which method performed better. To address this question, ideally there must first be a standard or “ground truth” data to compare results.

The VESGEN analysis produces colorized image maps with over 30 different quantitative data points. VESGEN is a user-friendly software already studied with FA and colonic vasculature imaging studies [5,20,21,30,31]. It has been through several revisions and upgrades, and the latest version released in 2021 (version 1.11) offers the most reliable and accurate output parameters. VESGEN generates parameters based on retinal vessel segmentation algorithms and physiological branching rules [32,33].

A drawback of VESGEN is that it requires hours of manual preparation preimage for analysis by the software. This meticulous process includes manually tracing retinal vessels, identifying arterial and venous characteristics, formatting images to appropriate input settings, along with implementing several checkpoints to ensure quality during the process. This time-consuming effort essentially precludes VESGEN as a feasible tool for clinical practice and is currently limited to research. This has resulted in the need for automated software to perform accurate vessel segmentation, avoiding the laborious manual preparation process. Moreover, color fundus vessel segmentation is a notoriously challenging image processing problem. Numerous papers have been published over four decades attempting to solve the problem with varying degrees of success [17,34].

We use a deep learning model to reduce processing time. Deep learning models are implicitly programmed, requiring a one-time training of around 100 h. Training examples consisting of pairs of CF images and their manually segmented vessels. During training, the model learns what combination of features are needed to result in correct segmentation. This shifts the bulk of image segmentation time to the training phase of the deep learning model, which would be done well prior to use in clinical practice. As a result, deep learning programming is expected to be more viable for clinical practice.

Our use of the deep learning model provides evidence of potential use in combination with VESGEN to become a feasible and highly sensitive tool for the visualization of the retina and could potentially be incorporated into clinical practice. While deep learning will speed up the process of image preparation, the ability to separate both arteries and veins was not available at the time of our study. Recently, as of July 2022, new insight into the possibility of separation is now available (https://github.com/rmaphoh/AutoMorph, accessed on 6 January 2020).

Binary images produced by deep learning are not comparable to manually segmented images for vessel tortuosity and area density of both total and artery-only. This is likely due to the lack of ability of deep learning to identify fine vessels, despite RVGAN being state-of-the-art for detecting fine vessels. It is probably that downscaling the images to STARE resolution made the finest vessels undetectable. Training RVGAN on a dataset with higher resolution than STARE might help with this issue. Regardless, deep learning showed the ability to identify similar structural changes in large vessels seen as fractal dimension (Df) across the retina compared to manual segmentation of CF images.

While this proof-of-concept study provides insight into the potential use of CF imaging in future studies of pulmonary vascular diseases, our study has the limitations of including a small number of subjects. VESGEN in conjunction with deep learning processing, has great potential for speeding the preparation of images and potentially making this approach feasible for future clinical use, specifically aiding in earlier disease detection while providing clinicians the ability for easy longitudinal monitoring of retinal vascular changes that are both objective and comparable for each subject. This combination may also improve patient safety by eliminating invasive imaging techniques, improving diagnostic power, and faster and cost-efficient examinations.

## 5. Conclusions

We identified several key advantages of utilizing CF paired with VESGEN analysis as a retinal imaging modality over FA with manual input of images. CF is a non-invasive imaging modality. It possesses the ability, alongside image processing segmentation, to provide valuable insight into vascular patterns of vessel generations 1–5 to understand systemic manifestations of PAH in the eye repeatedly and rapidly. One limitation of this study was the use of VESGEN manual preparation prior to program analysis. This time-consuming technique basically removes VESGEN as a viable clinical practice tool, relegating it to research use only. As a result, there is a growing demand for automated software that can execute exact vessel segmentation without the time-consuming human preparation step. Additional validation is needed before this can be introduced in a clinical setting.

## Data Availability

The data presented in this study are available on request from the corresponding author.
